# Natural Bioactive Compounds Exerting Health-Promoting Effects by Ameliorating Oxidative Stress

**DOI:** 10.3390/antiox14010085

**Published:** 2025-01-13

**Authors:** Bin Du, Baojun Xu

**Affiliations:** 1 Hebei Key Laboratory of Natural Products Activity Components and Function, Hebei Normal University of Science and Technology, Qinhuangdao 066004, China; bindufood@aliyun.com; 2 Guangdong Provincial Key Laboratory IRADS, Department of Life Sciences, BNU-HKBU United International College, Zhuhai 519087, China

Oxidative stress is caused by various intrinsic and extrinsic factors. It is a widely accepted notion that dietary or dietary supplement interventions can decrease levels of oxidative stress [1]. Natural bioactive compounds (such as flavonoids, stilbenes, terpenes, alkaloids, saponins, polysaccharide, etc.) have been reported to exhibit a multitude of health-promoting effects including antioxidant activity. Previous studies have shown that the mechanisms involved in the amelioration of oxidative stress include the regulation of GPX4 and ferroptosis [2]. However, the underlying cellular signaling and molecular mechanisms of their effect in ameliorating oxidative stress are not fully understood.

This Special Issue aims at collate and summarize the observed antioxidant activity of natural bioactive compounds (such as saponins, ergothioneine, flavonoids, taurine, chlorogenic acid, and phenolic compounds) and extracts (including bean extracts, *Moringa oleifera* extracts, Massa Medicata Fermentata, goji-berry extracts and dietary nitrate) based on studies from in vitro models (cell culture models; the gastrointestinal digestion model) and in vivo models (the *Caenorhabditis elegans* model and zebrafish model). It contains 11 papers covering the above-mentioned topics, including 1 systematic review and meta-analysis, and 10 research papers.

Flavonoids and phenolic compounds are a large group of plant metabolites that have long garnered interest from researchers worldwide due to their functions in human health and well-being [3]. Four contributions in this Special Issue address these compounds. In one, the bioactivity and stress resistance mechanisms of flavonoids derived from Chinese sea buckthorn leaves (such as FCL) were investigated. The authors identified six novel flavonoids. The results showed that FCL possesses potent antioxidant properties, as evidenced by its ability to effectively scavenge DPPH free radicals and demonstrate ferric-reducing antioxidant power and oxygen radical absorbance capacity levels comparable with Trolox. Furthermore, FCL showed remarkable efficacy in reducing reactive oxygen species (ROS) levels and malondialdehyde (MDA) levels while enhancing the activities of key antioxidant enzymes, namely superoxide dismutase and catalase in *C. elegans* (Contribution 1). The second contribution evaluates the effect of digestion on the phenolic compounds and antioxidant capacity of *Artocarpus heterophyllus* Lam. (jackfruit) pulp using an in vitro gastrointestinal digestion model. The results showed that the total phenol content (TPC) was significantly higher after gastric digestion. Thirty phenolic compounds (hydroxybenzoic acids and derivatives, hydroxycinnamic acids and derivatives, and flavonoids) were identified. The antioxidant activities of the digested samples varied with the TPC, and there was a correlation between antioxidant activity and TPC (Contribution 2). The third contribution tests cost-effective staple foods by fortifying them with *M. oleifera* leaf powder (MOLP) and its phenolic-rich extract. Moreover, the antioxidant, antibacterial, and antidiabetic properties of the phenolic extract from MOLP were observed. The resulting extract had a TPC of 138.2 mg of gallic acid equivalents/g. ABTS and DPPH assays presented IC_50_ values of 115.2 mg/L and 544.0 mg/L, respectively. Furthermore, the extract exhibited notable α-amylase inhibition and no cytotoxicity towards human fibroblasts (Contribution 3). Chlorogenic acid (CGA) is a widely distributed natural compound with many crucial pharmacological effects, including antioxidant, anti-inflammatory, antibacterial, antiviral, hypoglycemic, lipid lowering, anti-cardiovascular, antimutagenic, anticancer, and immunomodulatory effects [4]. Wang et al. assessed the effect and mechanisms of CGA in the alleviation of cytotoxicity induced by aflatoxin B1 (AFB1) in L-02 cells. These findings indicated that CGA (160 μM) significantly recovered cell viability and cell membrane integrity in AFB1-treated (8 μM) cells. Furthermore, it was found that CGA reduced AFB1-induced oxidative injury by neutralizing ROS and activating the nuclear factor erythroid 2-related factor 2 (Nrf2)/heme oxygenase-1 (HO-1) signaling pathway (Contribution 4).

A growing body of evidence indicates that legumes exhibit potent antioxidant properties that protect against various chronic diseases induced by oxidative stress [5]. In this Special Issue, a further two papers focus on bioactive bean extracts: Luo et al. further elucidated the anti-obesity effects of adzuki bean saponins (ABSs) in improving lipid metabolism and oxidative stress, exploring the effective ingredients and potential molecular mechanisms through UHPLC-QE-MS analysis, network pharmacology, bioinformatics, and in vitro experiments both in the 3T3-L1 cell line and HepG2 cell line. Their results showed that ABS can improve intracellular lipid accumulation, adipogenesis, oxidative stress, and mitochondrial damage caused by lipid accumulation including ROS generation, abnormal mitochondrial membrane potential, and ATP disorder (Contribution 5). In another contribution, eight bean extracts (BE1–BE8) were tested for their ability to inhibit lipid accumulation and oxidation in hepatocytes. The results indicated that the antioxidants BE2 and BE8 are potential candidates for the regulation of triacylglycerol (TAG) and TAG hydroperoxide (TGOOH) accumulation in fatty acid-induced lipid droplets (LDs) (Contribution 6).

In another contribution, the chondrocyte-protective functions of ergothioneine (EGT) were investigated using a model of oxidative stress-induced injury of chondrocytes by hydrogen peroxide (H_2_O_2_). Human chondrocytes were subjected to oxidative stress induced by H_2_O_2_ treatment; cell viability, the activity of lactate dehydrogenase (LDH) released into the medium, dead cell ratio, intracellular ROS production, and mitochondrial morphology were assessed. EGT improved chondrocyte viability and LDH activity in the medium and strongly suppressed the dead cell ratio. EGT also exerted protective effects on intracellular ROS production and mitochondrial morphology (Contribution 7).

Seol et al. explored the antioxidative and protective effects of taurine chloramine (Tau-Cl) in astrocytes. Tau-Cl increased the expression and nuclear translocation of nuclear factor E2-related factor (Nrf2) and the expression of Nrf2-regulated antioxidant genes, including heme oxygenase 1 (HO-1). They also found that Nrf2 activity is negatively regulated by Kelch-like ECH-associated protein 1 (Keap1). Tau-Cl decreased the level of the reduced thiol groups of Keap1, resulting in disruptions to the Keap1–Nrf2 complex (Contribution 8).

Furthermore, in an investigation of the antioxidant activity of Massa Medicata Fermentata (MMF), the ability of the hydrothermal extract of MMF to scavenge free radicals corresponding to biological oxidative stresses was demonstrated. In addition, the effects of MMF on the body mass index and cholesterol accumulation in adult zebrafish fed a high-cholesterol diet for three weeks were examined. MMF prevented DNA and lipid damage caused by oxidative stress, inhibited LDL oxidation, and reduced the expression of cytokines and related proteins (MAPK and NFκB), with prominent anti-oxidative pathway (NRF2-HO-1) activation properties (Contribution 9).

Li et al. employed a rapid and convenient method applicable in the green synthesis of gold nanoparticles (AuNPs) using goji berry (*Lycium barbarum*—LB) extracts. Moreover, they evaluated their structural analysis and evaluation of the induced antioxidant protection and anti-inflammatory effects of the synthesized gold nanoparticles in endothelial cells (HUVECs) exposed to hyperglycemia. The results of this study reveal that AuNPs with a predominantly spherical shape and an average size of 30 nm were obtained. The ultraviolet-visible spectrum showed a characteristic absorption band at λmax = 536 nm for the AuNPs. FTIR analysis revealed the presence of phenolic acids, flavonoids and carotenoids, acting as the capping and stabilizing agents of AuNPs. Both the *L. barbarum* extract and AuNPs were well tolerated by HUVECs, increased the antioxidant defense and decreased the production of inflammatory cytokines induced via hyperglycemia-mediated oxidative damage (Contribution 10).

Finally, this Special Issue includes one systematic review and meta-analysis of randomized controlled trials. Data from Liang et al. showed that the PRISMA guidelines were adhered to in the studies analyzed. MEDLINE and SPORTDiscus were searched from inception to January 2023. A paired analysis model for cross-over trials was incorporated to perform a random-effects meta-analysis for each performance outcome and to generate standardized mean differences (SMDs) between the inorganic nitrate (NO_3_^−^) and placebo supplementation conditions. This systematic review and meta-analysis included 27 and 23 studies, respectively. The time to reach peak power (SMD: 0.75, *p* = 0.02), mean power output (SMD: 0.20, *p* = 0.02), and total distance covered in the Yo-Yo intermittent recovery level 1 test (SMD: 0.17, *p* < 0.0001) were all improved after NO_3_^−^ supplementation (Contribution 11).

In summary, the results of the above-mentioned studies will help to address the challenges remaining in this field in terms of the limited knowledge about natural bioactive compounds and their underlying cellular signaling and molecular mechanisms. These studies may be potential resources for the development of functional foods and drug discovery.
